# *Helicobacter* *pylori* inhibits autophagic flux and promotes its intracellular survival and colonization by down‐regulating SIRT1

**DOI:** 10.1111/jcmm.16411

**Published:** 2021-02-28

**Authors:** Xin Wang, Bo Wang, Wei Gao, Yifei An, Guoying Dong, Jihui Jia, Qing Yang

**Affiliations:** ^1^ Institute of Pathogen Biology School of Basic Medical Sciences Cheeloo College of Medicine Shandong University Jinan China; ^2^ Department of Traditional Medicine Qilu Hospital Cheeloo College of Medicine Shandong University Jinan China; ^3^ Department of Pathology Jinan Central Hospital Cheeloo College of Medicine Shandong University Jinan China; ^4^ Key Laboratory for Experimental Teratology of Ministry of Education Shandong University Jinan China; ^5^ Shandong Key Laboratory of Infection and Immunity Shandong University Jinan China; ^6^ Karolinska Institute Collaborative Laboratory for Cancer research Shandong University Jinan China

**Keywords:** autophagic flux, autophagosome, *Helicobacter pylori*, intracellular colonization, intracellular survival, SIRT1

## Abstract

*Helicobacter pylori* (*H. pylori*) is the strong risk factor for a series of gastric pathological changes. Persistent colonization of *H. pylori* leading to chronic infection is responsible for gastritis and malignancy. Autophagy is an evolutionary conserved process which can protect cells and organisms from bacterial infection. Here, we demonstrated that *H. pylori* infection induced autophagosome formation but inhibited autophagic flux. SIRT1, a class III histone deacetylase, was down‐regulated at both mRNA and protein levels by *H. pylori* infection in gastric cells. Further investigation showed that the transcriptional factor RUNX3 accounted for down‐regulation of SIRT1 in *H. pylori*‐infected gastric cells. SIRT1 promoted autophagic flux in gastric cells and activation of SIRT1 restored the autophagic flux inhibited by *H. pylori* infection. Furthermore, SIRT1 exerted inhibitory effects on intracellular survival and colonization of *H. pylori*. And activation of autophagic flux in SIRT1‐inhibited gastric cells could significantly reduce intracellular load of *H. pylori*. Moreover, the relationship between *H. pylori* infection and SIRT1 expression was identified in clinical specimen. Our findings define the importance of SIRT1 in compromised autophagy induced by *H. pylori* infection and bacterial intracellular colonization. These results provide evidence that SIRT1 can serve as a therapeutic target to eradicate *H. pylori* infection.

## 1 INTRODUCTION

*Helicobacter pylori* (*H. pylori*) is one of the most common human pathogens that has colonizes more than 50% of the world’s population. Chronic infection of *H. pylori* gives rise to a series of well‐defined histological stages processing through gastritis, metaplasia, dysplasia and eventually the malignancy, gastric cancer.^1^ Although *H. pylori* was previously regarded as an extracellular bacterium, a growing evidence has reported the facultative intracellular nature of *H. pylori*.^2^, ^3^ This bacterium was found to survive and even multiply inside gastric epithelial cells.^3^, ^4^ Presence of intracellular *H. pylori* in progenitor or stem cells serves as a strategy for this invasive pathogen to persist in human stomach.^5^, ^6^ Hiding inside the host cells enables *H. pylori* to escape from the innate immune response.^7^ Besides, intracellular *H. pylori* are more resistant to antibiotic treatment and account for repopulation following eradication therapy.^2^, ^8^, ^9^ However, the host factors that affect the intracellular survival and colonization of *H. pylori* remain poorly understood.

Autophagy is an evolutionary conserved process which may be divided into two steps. First, double‐membrane autophagosomes formed sequestrating intracellular components. Second, autophagosome‐lysosome fused and eventually degraded the contents. The second step is also named as autophagic flux. Autophagy can be induced by a number of deleterious stimuli and play an important role in maintaining cellular homeostasis and protecting against pathogen infection.^10^, ^11^ Intracellular bacteria like *Mycobacterium tuberculosis* (*M*. *tuberculosis*) and *Salmonella enterica* may evade degradation by autophagy through different mechanisms thereby leading to persistent infection and increased bacterial load. The pathogens may obstruct phagosomal lipid metabolism to inhibit autophagosome maturation or escape from the host cell autophagosomes.^12^, ^13^ It has been reported that *H. pylori* can induce autophagosomes formation, survive and even multiply in autophagosomes before autophagic flux occurs.^14^, ^15^ When autophagosomes fused with lysosome, which is referred to autophagic flux, clearance of *H. pylori* in a lysosome‐dependent manner occurred. Therefore, inhibiting autophagic flux may increase intracellular survival and colonization of *H. pylori*.^15^ Nevertheless, the mechanism by which *H. pylori* antagonizes degradation by autophagic flux remains to be elucidated.

Sirtuin 1 (SIRT1) belongs to class III histone deacetylases and is the mammalian ortholog of yeast silent information regulator (Sir 2). As Sir 2 was proved to extend lifespan in yeast, research on SIRT1 first concentrated on anti‐ageing and then expanded to stress response and metabolism.^16^, ^17^, ^18^ By interacting with the energy sensor, AMP‐activated protein kinase, SIRT1 was shown to inhibit mammalian target of rapamycin (mTOR) signalling and thus promote formation of autophagosomes.^19^ SIRT1 can also directly deacetylate ATGs and BECLIN1 and stimulates the initial process of autophagy.^20^, ^21^ Moreover, SIRT1 deacetylates and activates transcription factor FOXO1 and its target Rab7, which was both necessary and sufficient for increasing autophagic flux.^22^ Moreover, SIRT1‐dependent autophagy activation was found to reduce bacterial growth and anti‐*M*. *tuberculosis* responses.^21^, ^23^ However, whether SIRT1 and SIRT1‐induced autophagy play a role in intracellular survival and colonization of *H. pylori* has not been elucidated.

In this study, we investigated regulation of SIRT1 expression by *H. pylori* infection and the underlying mechanism was explored in detail. Then the effects of SIRT1 and SIRT1‐induced autophagic flux in intracellular survival and colonization of *H. pylori* were also explored.

## 2 MATERIAL AND METHODS

### 2.1 Cell culture and reagents

Human gastric cell lines AGS, BGC‐823, SGC‐7901 and GES‐1 were obtained from Cell Resource Center, Institute of Biochemistry and Cell Biology at the Chinese Academy of Sciences (Shanghai, China) and cultured in F12 (AGS) or RPMI 1640 (BGC‐823, SGC‐7901 and GES‐1) with 10% foetal bovine serum (Biological Industries, Israel), 100 units/ml penicillin and 100 µg/ml streptomycin. All cells were incubated at 37°C with 5% CO_2_. Mycoplasma PCR (GeneCopoeia) testing of these cells was performed every month following the protocol of the manufacturer. Reagents used in this study were: Earle’s Balanced Salt Solution (EBSS, HyClone) used for serum starvation to induce autophagic flux, bafilomycin A_1_ (Baf A1, S1413, Selleckchem) used as inhibitor of autophagic flux, SRT1720 (S1129, Selleckchem) used as activator of SIRT1, and EX 527 (S1541, Selleckchem) used as inhibitor of SIRT1.

### 2.2 Bacterial culture

*H. pylori* strains (NCTC 26695 and 11637) were kindly provided by Dr. Jianzhong Zhang (Chinese Disease Control and Prevention Center; Beijing, China). These *H. pylori* strains were cultured on the Brucella broth plates containing 5% FBS under microaerophilic conditions (5% O_2_, 10% CO_2_, and 85% N_2_) at 37 °C. *H. pylori* strains were harvested, resuspended with PBS and added to gastric cells at a multiplicity of infection (MOI) of 100.

### 2.3 Plasmids and siRNAs

The pBabe‐myc‐RUNX3‐R122C plasmid (a mutation located in the conserved Runt domain) was kindly provided by Professor Bae SC (Chungbuk National University, Cheongju 361‐763, South Korea). The RUNX3 eukaryotic expression plasmid (pcDNA3.1‐RUNX3) was kindly provided by Dr. Zhifang Liu (Shandong University, Jinan, China). The luciferase reporter plasmids containing the promoter sequence of SIRT1 assumed RUNX3 binding sits deleted promoter sequences of SIRT1 were constructed by BioSune Biotechnology (Shanghai, China). The plasmids were delivered into cells by X‐tremeGENE HP Transfection Reagent (Roche Applied Science, Basel, Switzerland). The small interfering RNA (siRNA) targeting RUNX3 and FOXO3a, and the negative control siRNA were synthesized by GenePharma (Shanghai, China) and delivered into cells by Lipofectamine 2000 (Invitrogen). The sequences of siRNAs were summarized in Table S1.

### 2.4 RNA extraction and quantitative real‐time PCR (qRT‐PCR)

Total RNA was extracted with Trizol, converted into cDNA and amplified by qRT‐PCR as previously described.^24^ Primers were synthesized by BioSune Biotechnology, and the primer sequences were listed in Table S1.

### 2.5 Western blot

Total proteins were extracted with RIPA lysis buffer (Beyotime, Shanghai, China) containing proteinase inhibitor (PMSF, Beyotime). The protein concentrations were measured using a BCA reagent kit (Beyotime). The membranes were probed, and the protein bands were visualized as previously described.^24^ The primary antibodies used include the following: anti‐β‐actin (4967, 1: 1000, Cell Signaling), anti‐GAPDH (60004‐1, 1: 5000, Proteintech), anti‐LC3B (3868, 1: 500, Cell Signaling), anti‐p62 (ab56416, 1: 2000, Abcam), anti‐RUNX3 (ab135248, 1: 1000, Abcam) and anti‐SIRT1 (ab110304, 1: 5000, Abcam). Housekeeping gene β‐actin or GAPDH served as the internal control.

### 2.6 Luciferase assay

For luciferase assay, cells were seeded in 24‐well plates (5 × 10^4^ cells per well). After transfection with plasmids or siRNAs, appropriate reporter plasmid was transfected into cells. Then, the relative luciferase activities were measured and calculated using the Dual‐Luciferase Reporter Assay System (Promega).^24^

### 2.7 Chromatin immunoprecipitation (ChIP)

With the SimpleChIP^®^ Enzymatic Chromatin IP Kit (Cell Signaling), ChIP assays were performed with anti‐RUNX3 (ab11905, Abcam), and IgG (negative control) in AGS cells transfected with pcDNA3.1‐RUNX3 plasmid. The purified DNA served as the template to amplify SIRT1 promoter. Agarose gel electrophoresis was performed to visualize the PCR products. The primers were listed in Table S1.

### 2.8 The mCherry‐EGFP‐LC3B fluorescence microscopy assay

Cells were seeded on glass coverslips in 24‐well plates (5 × 10^4^ cells per well) and incubated overnight. Then cells were transfected with AdM‐CMV‐mCherry‐EGFP‐LC3B adenovirus (MOI = 100, Vigene, Jinan, China) for 24 h. After cells were treated with chemicals or infected with *H. pylori*, cells were fixed with 4% fixative solution and the coverslips were taken out of the plates for observation. Over 20 cells in one field were randomly selected to examine the numbers of autophagosomes (yellow puncta) and autolysosomes (red puncta) using an Olympus microscope (Tokyo, Japan). Five fields were randomly selected from each group, and the EGFP/mCherry co‐localization efficiency was calculated.

### 2.9 PCR quantitation of bacterial DNA

DNA from gastric cells was extracted using a DNA purification kit (DP302, TIANGEN, Beijing, China) according to the manufacturer’s instruction. *H*. *pylori*‐specific 16S ribosomal DNA (rDNA) was quantified by real‐time PCR using *TransStart* Top Green qPCR SuperMix. The primer sequences were listed in Table S1. The levels of 16S rDNA in gastric cells were measured and normalized to *β‐actin* based on 2^‐ΔΔCt^ method.

### 2.10 Gentamicin protection assay

Cells were infected with *Hp26695* for 12 hours. To remove the nonadherent bacteria, cells were washed three times using warm PBS. Subsequently, the cell monolayers were incubated with gentamicin (E003632, 100 mg/ml, Sigma, St. Louis, MO) for 1 hours at 37°C, washed three times with warm PBS, and then treated with 0.5% saponin (HY‐100597, dissolved in PBS, Sigma) at 37°C for 15 minutes. The treated monolayers were resuspended thoroughly, serially diluted (1:30, 1:100, 1:300, 1:900, 1:2700) and plated on Brucella broth containing 10% FBS. After 5 days of incubation, the colony formation of intracellular bacteria was calculated as CFU per well of cells.

### 2.11 Immunofluorescence stain

Cells (2.5 × 10^4^) were seeded onto coverslips in 24‐well plates and incubated overnight. After infected with *Hp26695* for 10 h, extracellular *H. pylori* were killed using 100 µg/ml gentamicin for 1 hour at 37°C and then washed with PBS two times. Next, cells were fixed with 4% fixative solution and permeabilized with 0.2% Triton X‐100. Then, after blocking, cells were incubated with anti‐*H. pylori* antibody (ab7788, 1: 100, Abcam) and the fluorescent secondary antibody. Nuclei were stained with 4’, 6‐diamidino‐2‐phenylindole (DAPI, Beyotime). Slides were observed under microscope (Olympus), and photographs were taken using CellSens Dimension software. Red fluorescence intensities for the *H. pylori*‐infected group were calculated by randomly selecting over 30 cells in one field, and three fields were detected for each group (Image J).

### 2.12 Immunohistochemistry (IHC) stain

We obtained paraffinized gastric tissue sections from Jinan Central Hospital. *H. pylori* infection was examined by rapid urease test and pathological examination using methylene blue staining. These paraffinized gastric tissue sections were divided into two groups: *H. pylori*‐positive (n = 15) and *H. pylori*‐negative (n = 15). For each group, there were three types of pathological changes: superficial gastritis (n = 5), atrophic gastritis (n = 5) and dysplasia (n = 5). To investigate SIRT1 expression levels in these sections, IHC analysis was performed. Paraffin‐embedded sections were deparaffinized, rehydrated and antigen‐retrieved. The remaining steps were performed as previously described.^24^ The primary antibody anti‐SIRT1 (ab32441, 1: 100, Abcam) was used. The images were obtained under microscope (Olympus) using CellSens Dimension software. The intensity of SIRT1 staining was scored as follows: 0, no staining; 1, light brown; 2, medium brown; and 3, dark brown. The expression score was calculated by multiplying the staining intensity score and the positive percentages.

### 2.13 Statistical analysis

All experimental data were analysed with GraphPad Prism 8.0 software. Data were presented as mean ± SD. Comparisons between different groups were determined by Student’s t test or one‐way ANOVA. A value of *P* < 0.05 was considered statistically significant.

## 3 RESULTS

### 3.1 *H. pylori* infection induces autophagosome formation but inhibits autophagic flux leading to increased colonization

**FIGURE 1:**
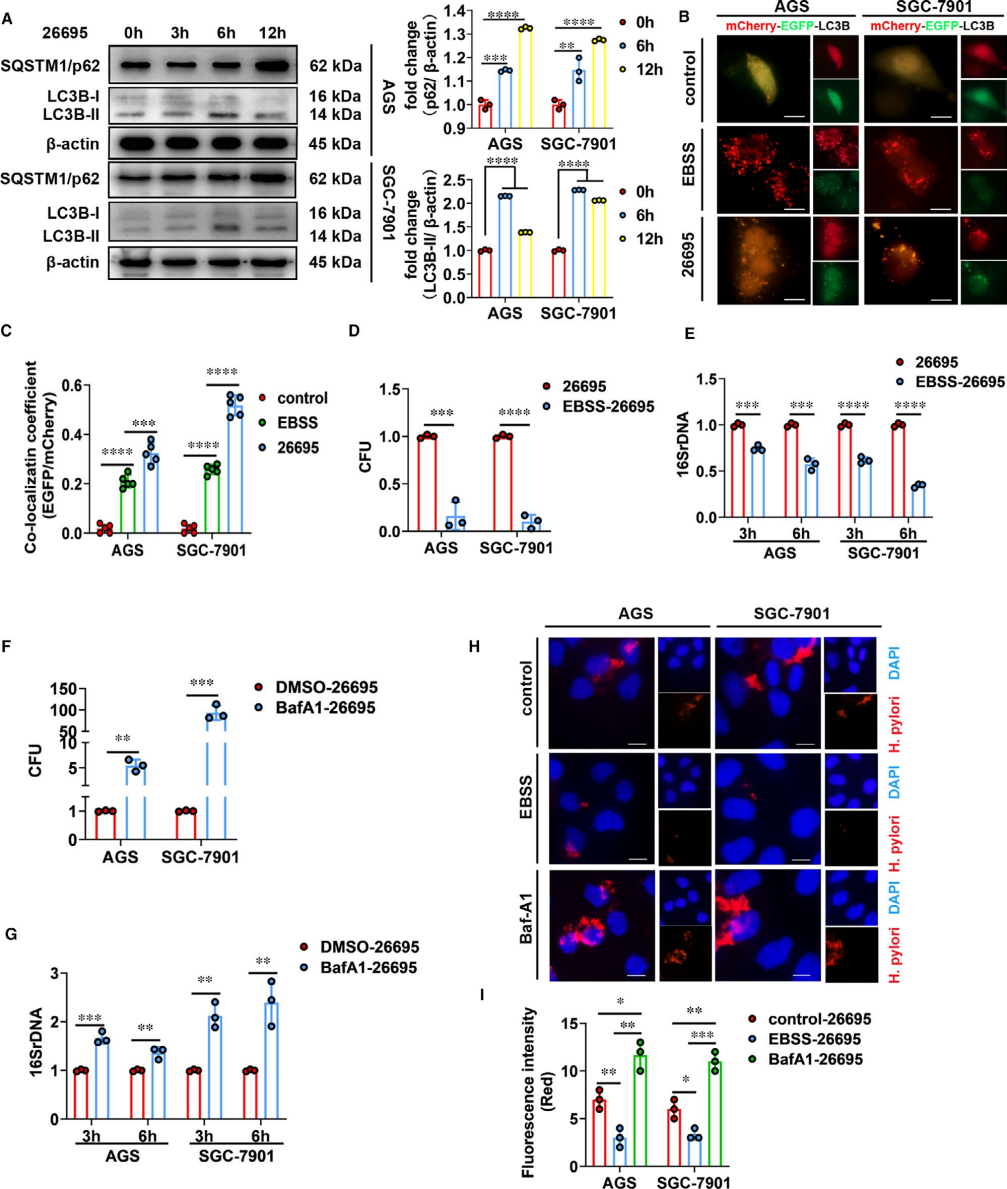
*H. pylori* infection induces autophagosome formation but inhibits autophagic flux leading to increased colonization *in vitro*. A, Western blot was performed to detect the protein levels of LC3BI/II and SQSTM1/p62 in cells infected with *Hp26695* at an MOI of 100 for indicated time. Data from 3 independent experiments are presented as mean ± SD. B and C, The mCherry‐EGFP‐LC3B fluorescence microscopy assays were performed in cells treated with EBSS (3 h, to induce autophagic flux) or infected with *Hp26695* (3 h). Data from 5 independent experiments are presented as mean ± SD. Scale bars: 10 µm. D–G, Colony formation assays (D and F) and bacterial DNA quantitation (E and G) were performed after treating *Hp26695*‐infected cells with EBSS (12 h) or Baf A1 (10 nM, 1 h, an inhibitor of autophagic flux). Data from 3 independent experiments are presented as mean ± SD. (H and I) Immunofluorescence staining of *H. pylori* after treating *Hp26695*‐infected cells with EBSS (12 hr) or Baf A1 (10 nM, 1 hr). Data from 3 independent experiments are presented as mean ± SD. Scale bars: 10 µm. *represents *P* < 0.05, **represents *P* < 0.01, *** represents *P* < 0.001 and **** represents *P* < 0.0001

We first verified increased autophagosome formation induced by *H. pylori* infection (*Hp26695* and *Hp11637*). As revealed in Figure 1A and Figure S1A,B, *H. pylori* infection significantly increased protein levels of LC3B‐II (a hallmark of autophagy) in gastric cell lines (AGS and SGC‐7901). Additionally, another autophagy marker, SQSTM1/p62 was also examined. Similar to LC3B‐II, protein levels of SQSTM1/p62 significantly increased in *H. pylori*‐infected gastric cell lines (Figure 1A, Figure S1A,B). Concomitantly increased levels of both LC3B‐II and SQSTM1/p62 indicate that autophagic flux is impaired by *H. pylori* infection. The impairment of autophagic flux induced by *H. pylori* infection was further confirmed by expressing mCherry‐EGFP‐LC3B fusion protein in AGS and SGC‐7901 cells. Fluorescence from EGFP but not mCherry is quenched in late acidic autophagosomes, so autophagic flux appears as red dots. For the early autophagosomes, EGFP signals overlap with mCherry and appear as yellow dots. Using this method, we found that in cells treated with EBSS (serum starvation to induce autophagic flux), most of the autophagosomes retained only mCherry signals indicating autophagic flux occurred (Figure 1B,C, Figure S1C,D). In contrast, in cells infected with *H. pylori*, we found increased retention of EGFP signals (yellow dots) and very few single mCherry signals (red dots) indicating compromised autophagy in *H. pylori*‐infected cells (Figure 1B,C).

Then, we assessed the effects of autophagic flux on intracellular survival and colonization of *H. pylori*. Colony formation assays, bacterial rDNA quantitation and immunofluorescence staining of *H. pylori* were performed to evaluate intracellular load of *H. pylori*. Results from the above assays revealed that inducing autophagic flux using EBSS decreased colonization of *H. pylori* whereas compromised autophagy induced by Baf A1 (an inhibitor of autophagic flux) exerted the opposite effects (Figure 1D‐I). Taken together, we confirmed that *H. pylori* infection inhibited autophagic flux which led to increased intracellular survival and colonization.

### 3.2 *H. pylori* infection inhibits expression levels of SIRT1 in gastric cells

**FIGURE 2:**
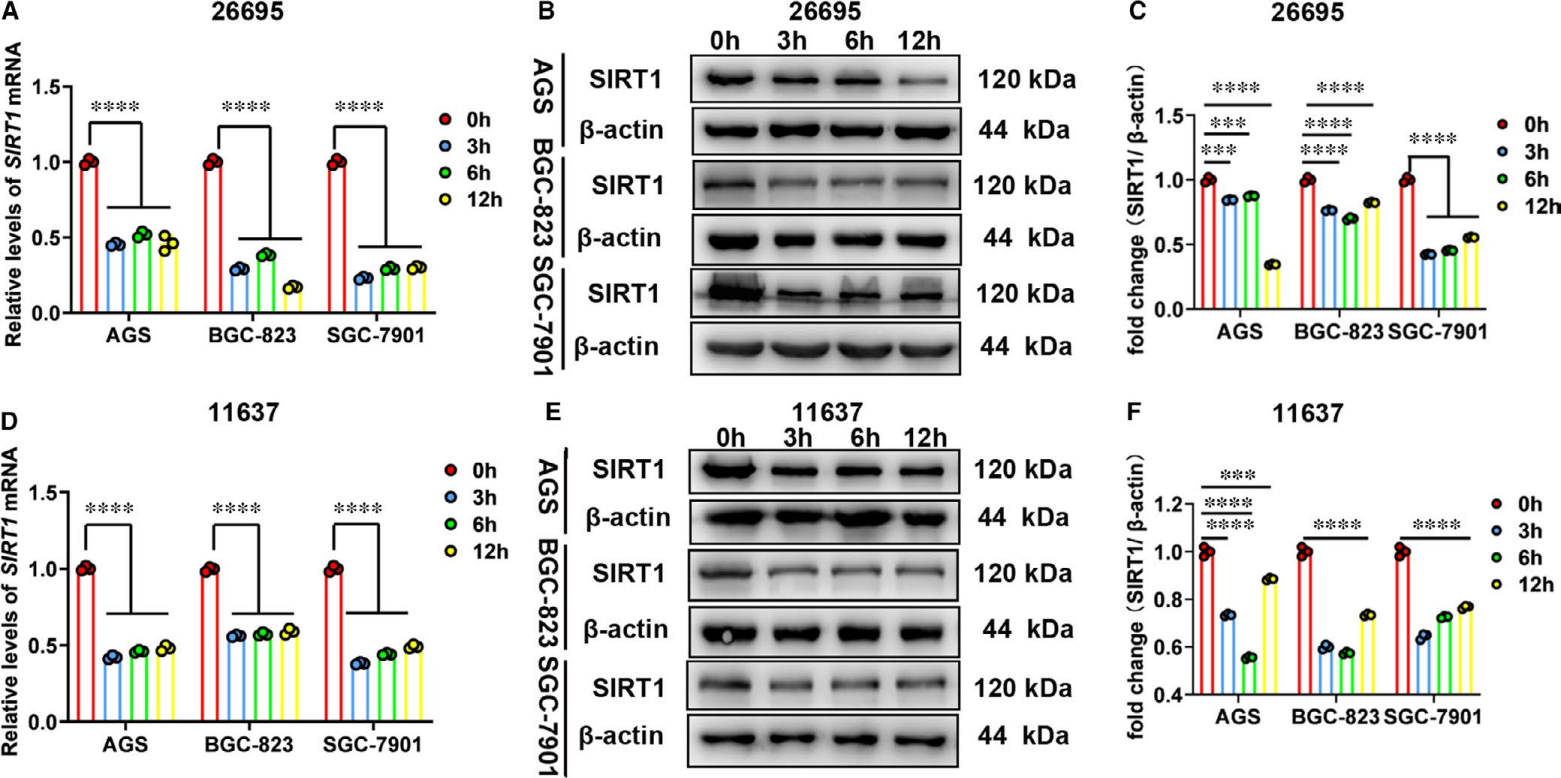
*H. pylori* infection inhibits expression levels of SIRT1 in gastric cells. A, The qRT‐PCR analysis of SIRT1 mRNA levels in cells infected with *Hp26695*. B and C, Western blot analysis of SIRT1 protein levels in cells infected with *Hp26695*. D, The qRT‐PCR analysis of SIRT1 mRNA levels in cells infected with *Hp11637*. E and F, Western blot analysis of SIRT1 protein levels in cells infected with *Hp11637*. Data from 3 independent experiments are presented as mean ± SD. ***represents *P* < 0.001 and **** represents *P* < 0.0001

Since SIRT1 can activate autophagy to eliminate bacterial load,^21^, ^23^ we have been suggested that SIRT1 might be the downstream target of *H. pylori* infection and play a role in intracellular survival and colonization of *H. pylori*. Then, we attempted to determine whether SIRT1 levels could be affected by *H. pylori* infection in several gastric cell lines. The qRT‐PCR and Western blot analysis were performed to examine levels of SIRT1, and two different *H. pylori* strains (*Hp26695* and *Hp11637*) were used to infect gastric cells. We found that SIRT1 was significantly down‐regulated at both mRNA and protein levels in *H. pylori*‐infected cells (Figure 2, Figure S2).

### 3.3 RUNX3 can regulate expression of SIRT1 in gastric cells

**FIGURE 3:**
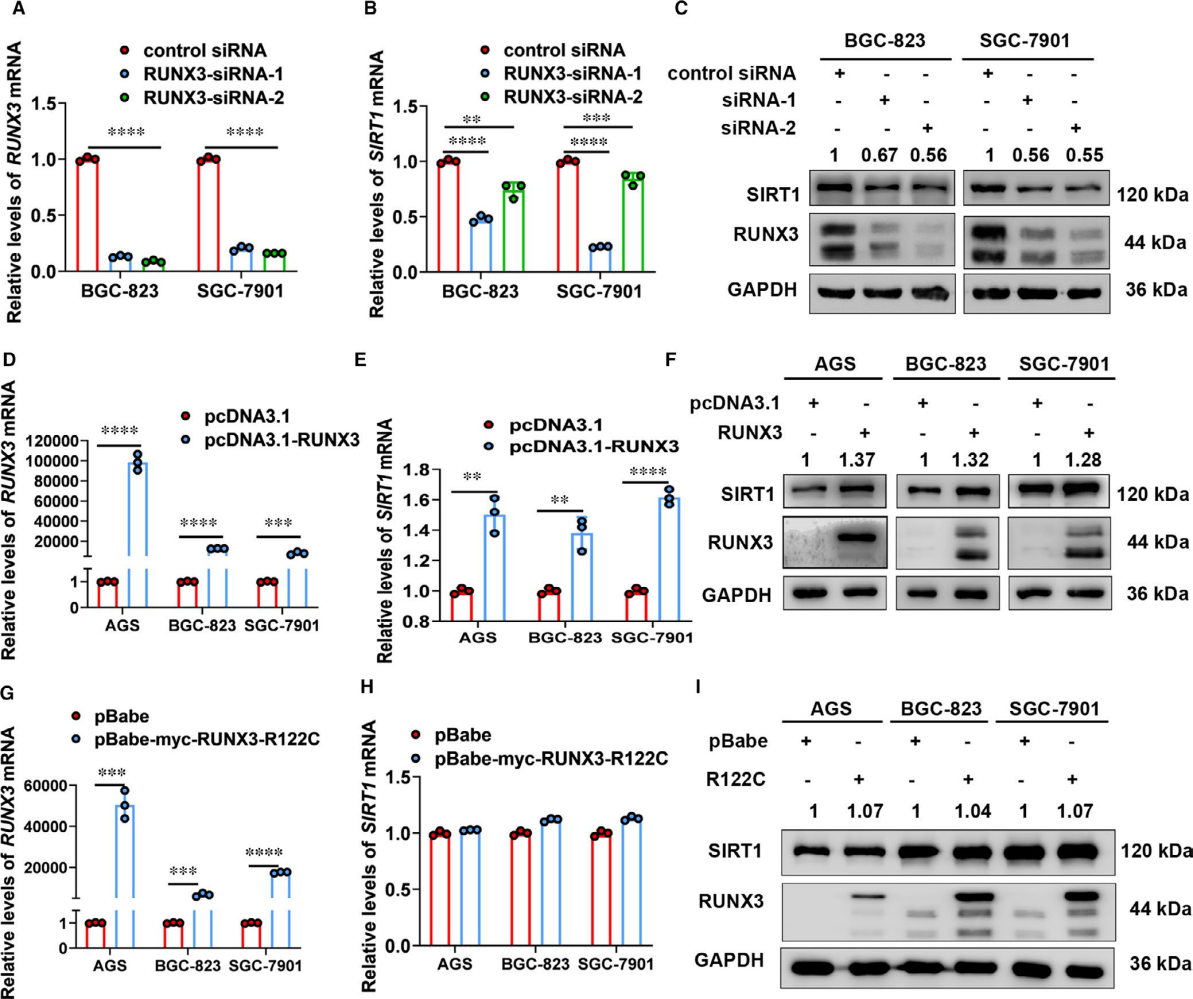
Regulation of SIRT1 expression by RUNX3 in gastric cells. A and B, The qRT‐PCR analysis of RUNX3 (A) and SIRT1 (B) mRNA levels in cells transfected with siRNAs targeting RUNX3. Data from 3 independent experiments are presented as mean ± SD. C, Western blot analysis of RUNX3 and SIRT1 protein levels in cells transfected with siRNAs targeting RUNX3. The mean values are indicated (n = 3). D and E, The qRT‐PCR analysis of RUNX3 (D) and SIRT1 (E) mRNA levels in cells transfected with RUNX3‐expressing vector. Data from 3 independent experiments are presented as mean ± SD. F, Western blot analysis of RUNX3 and SIRT1 protein levels in cells transfected with RUNX3‐expressing vector. The mean values are indicated (n = 3). G and H, The qRT‐PCR analysis of RUNX3 (G) and SIRT1 (H) mRNA levels in cells transfected with Runt domain mutant RUNX3‐expressing vector. Data from 3 independent experiments are presented as mean ± SD. I, Western blot analysis of RUNX3 and SIRT1 protein levels in cells transfected with Runt domain mutant RUNX3‐expressing vector. The mean values are indicated (n = 3). ** represents *P* < 0.01, *** represents *P* < 0.001 and **** represents *P* < 0.0001

**FIGURE 4:**
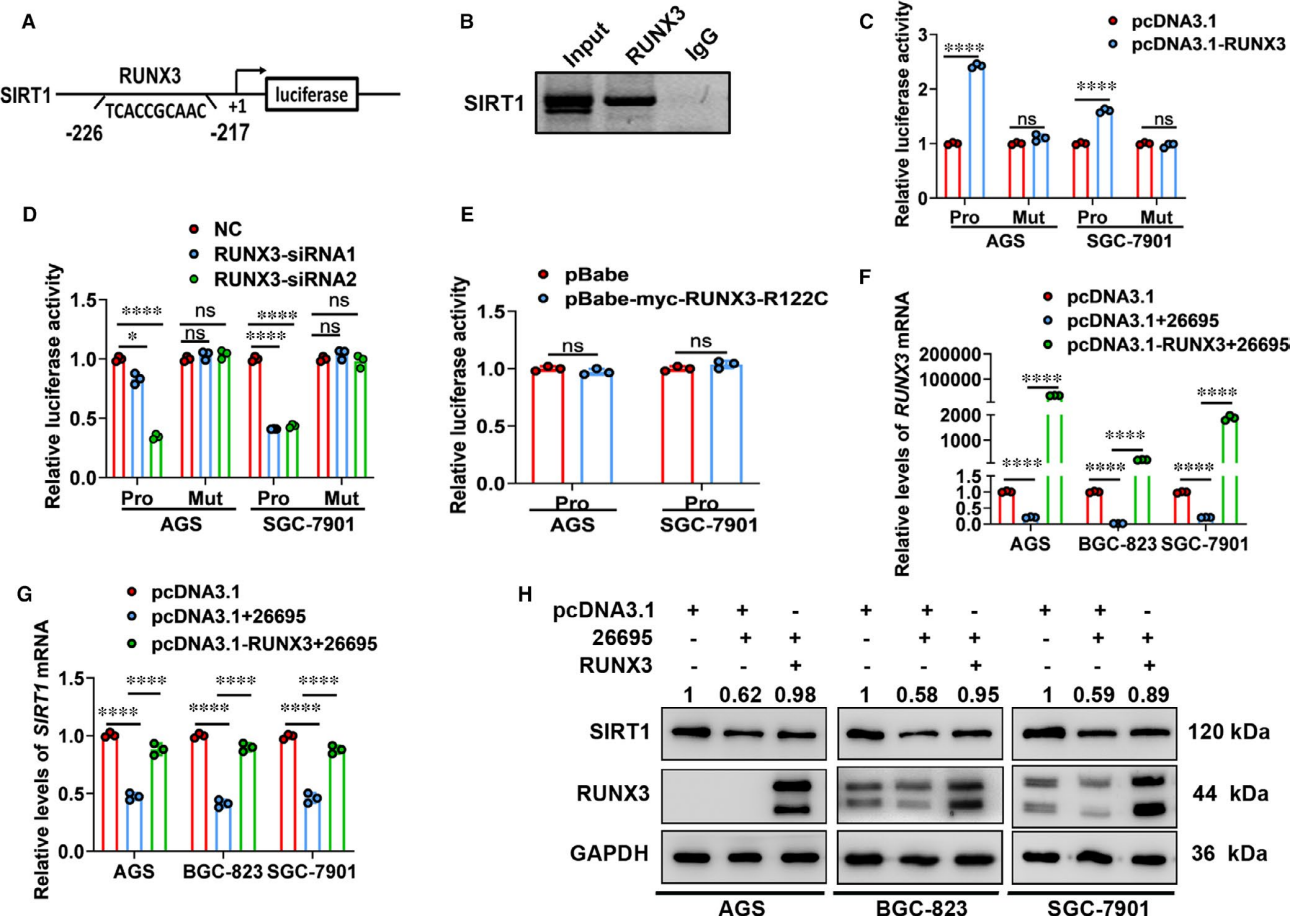
RUNX3 accounts for down‐regulation of SIRT1 in *H. pylori*‐infected gastric cells. A, The scheme of the putative RUNX3‐binding site in the SIRT1 promoter region. B, ChIP assay of RUNX3 directly binding to the promoter of SIRT1 (n = 3). C–E, Luciferase activities of different SIRT1 promoter constructs in cells transfected with RUNX3‐expressing vector (C), siRNAs targeting RUNX3 (D) or Runt domain mutant RUNX3‐expressing vector (E). Data from 3 independent experiments are presented as mean ± SD. F and G, The qRT‐PCR analysis of RUNX3 (F) and SIRT1 (G) mRNA levels in gastric cells transfected with RUNX3‐expressing vector and infected with *Hp26695* for 3 h. Data from 3 independent experiments are presented as mean ± SD. H, Western blot analysis of RUNX3 and SIRT1 protein levels in gastric cells transfected with RUNX3‐expressing vector and infected with *Hp26695* for 3 h. The mean values are indicated (n = 3). * represents *P* < 0.05 and **** represents *P* < 0.0001

Next, we screened for transcriptional factors responsible for regulation of SIRT1 expression in gastric cells. We searched the promoter of SIRT1 in JASPAR database for transcriptional factors that are closely related to *H. pylori* infection and found putative binding sites of FOXO3 and RUNX3 (Figure 4A, Figure S3A). Our results from small interference experiments showed that FOXO3 did not regulate SIRT1 expression in gastric cells (Figure S3B,C). In contrast, cells with RUNX3 knockdown showed significantly decreased SIRT1 expression (Figure 3A‐C). To further confirm regulation of SIRT1 by transcriptional factor RUNX3, we transfected cells with the RUNX3‐expressing vector (pcDNA3.1‐RUNX3) and the mutant vector (pBabe‐myc‐RUNX3‐R122C, the mutation located in the conserved Runt domain), respectively. Results from qRT‐PCR and Western blot analysis showed that overexpression of RUNX3 up‐regulated expression of SIRT1 whereas expression of mutant RUNX3 did not exert this positive regulation in gastric cells (Figure 3D‐I). These results suggested that RUNX3 can regulate expression of SIRT1 in gastric cells and this regulation is Runt domain‐required.

Furthermore, we performed ChIP assays in RUNX3‐expressing vector‐transfected AGS cells and demonstrated direct occupancy of RUNX3 on SIRT1 promoter (Figure 4B). Additionally, luciferase assays were used to verify the functional significance of this binding. We constructed luciferase reporter plasmids containing SIRT1 intact promoter (Pro) and mutant SIRT1 promoter (Mut). Overexpression of RUNX3 significantly increased promoter activities, while inhibition of RUNX3 exhibited the opposite effects (Figure 4C,D). Nevertheless, mutation of RUNX‐binding site abolished the changes in promoter activities (Figure 4C,D). Moreover, expression of mutant RUNX3 did not regulate SIRT1 promoter activities (Figure 4E). Therefore, in gastric cells SIRT1 was a direct target gene of RUNX3.

### 3.4 RUNX3 accounts for down‐regulation of SIRT1 in *H. pylori*‐infected gastric cells

Then, we assessed whether RUNX3 was involved in SIRT1 expression inhibited by *H. pylori* infection. As shown in Figure 4F‐H, *Hp26695* infection decreased expression of transcriptional factor RUNX3 and its target gene SIRT1 at both mRNA and protein levels. In RUNX3‐overexpressing gastric cells, expression of RUNX3 increased significantly even upon *Hp26695* infection. And overexpression of RUNX3 rescued expression of SIRT1 in gastric cells infected with *Hp26695*. Taken together, *H. pylori* infection inhibited expression of SIRT1 in gastric cells in a RUNX3‐dependent manner.

### 3.5 Activation of SIRT1 rescues autophagic flux inhibited by *H. pylori* infection

**FIGURE 5:**
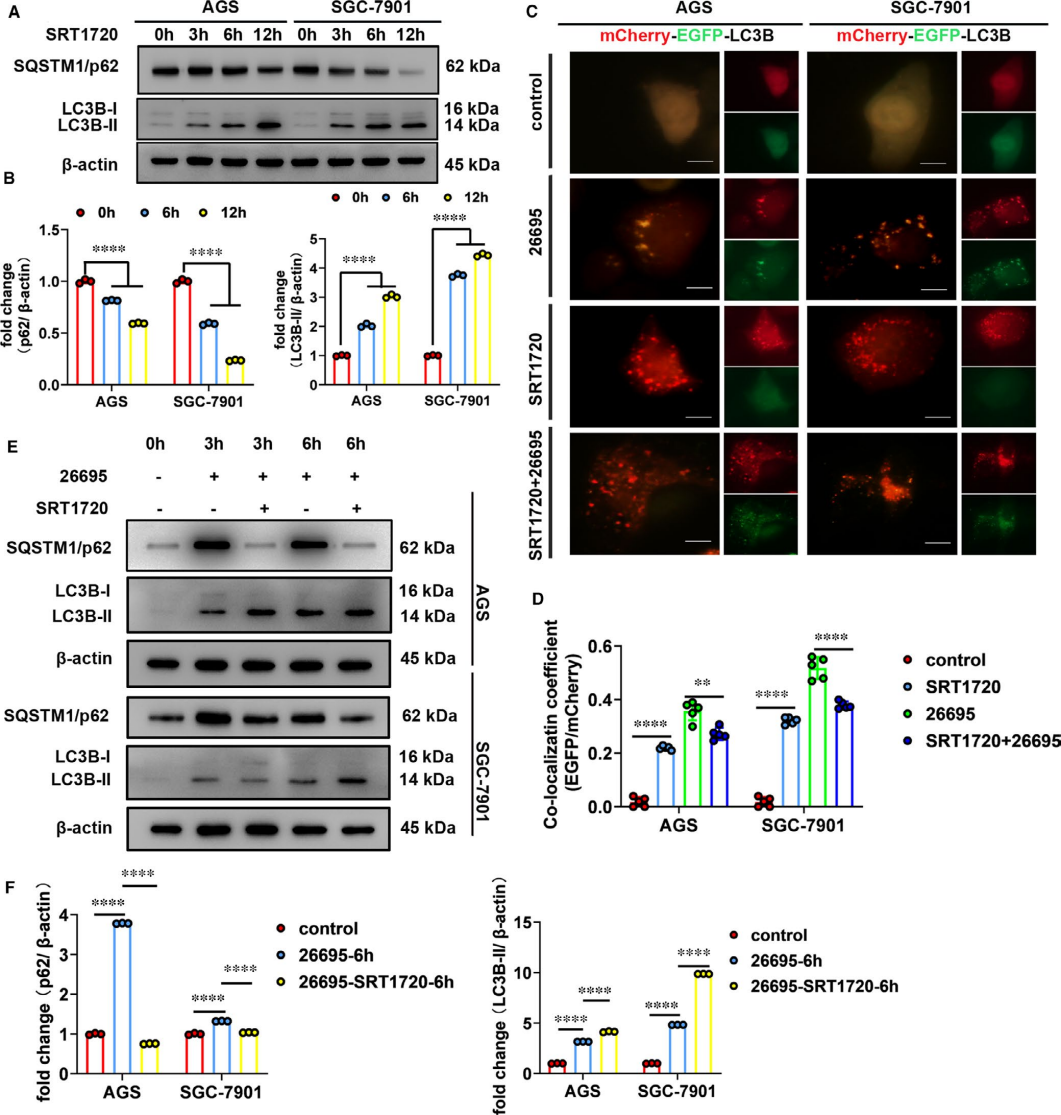
Activation of SIRT1 rescues autophagic flux inhibited by *H. pylori* infection. A and B, Western blot was performed to detect the protein levels of LC3BI/II and SQSTM1/p62 in cells treated with SIRT1 activator, SRT1720 (5 µM) for indicated time. Data from 3 independent experiments are presented as mean ± SD. C and D, The mCherry‐EGFP‐LC3B fluorescence microscopy assay. Cells were infected with *Hp26695* (3 h), or treated with SRT1720 (5 µM, 3 h) or pretreated with SRT1720 (5 µM, 1 h) and then infected with *Hp26695* (3 h). Data from 5 independent experiments are presented as mean ± SD. Scale bars: 10 µm. E and F, Western blot was performed to detect the protein levels of LC3BI/II and SQSTM1/p62 in cells infected with *Hp26695* for indicated time. Before *Hp26695* infection, cells were pretreated with or without SRT1720 (5 µM, 1 h). Data from 3 independent experiments are presented as mean ± SD. ** represents *P* < 0.01 and **** represents *P* < 0.0001

Next, we tested whether increasing SIRT1 activity could restore the autophagic flux inhibited by *H. pylori* infection. To this end, we activated SIRT1 using SRT1720 (a small molecule compound specific to activate SIRT1) and assessed autophagic flux by detecting levels of autophagy marker and the mCherry‐EGFP‐LC3B fluorescence microscopy assays. We showed that upon SIRT1 activation, protein levels of LC3B‐II increased but SQSTM1/p62 decreased over time indicating induction of autophagic flux (Figure 5A,B). In addition, cells with SIRT1 activation showed that most of the LC3B‐positive autophagosomes lost EGFP signal but retained mCherry signal (Figure 5C,D). This is consistent with the results from serum‐starved cells shown in Figure 1B and confirmed that activation of SIRT1 leads to increased autophagic flux. Then, we investigated the effects of SIRT1 activator on autophagic flux in *H. pylori*‐infected cells. Following SRT1720 administration, *H. pylori*‐infected cells showed decrease in SQSTM1/p62 protein levels and less EGFP‐mCherry overlapping LC3B‐positive puncta (Figure 5C‐F). This indicated that autophagic flux in *H. pylori*‐infected cells was restored by SRT1720. Therefore, these data indicated that activation of SIRT1 rescued autophagic flux that was inhibited by *H. pylori* infection.

### 3.6 SIRT1 inhibits intracellular colonization of *H. pylori* by activating autophagic flux

**FIGURE 6:**
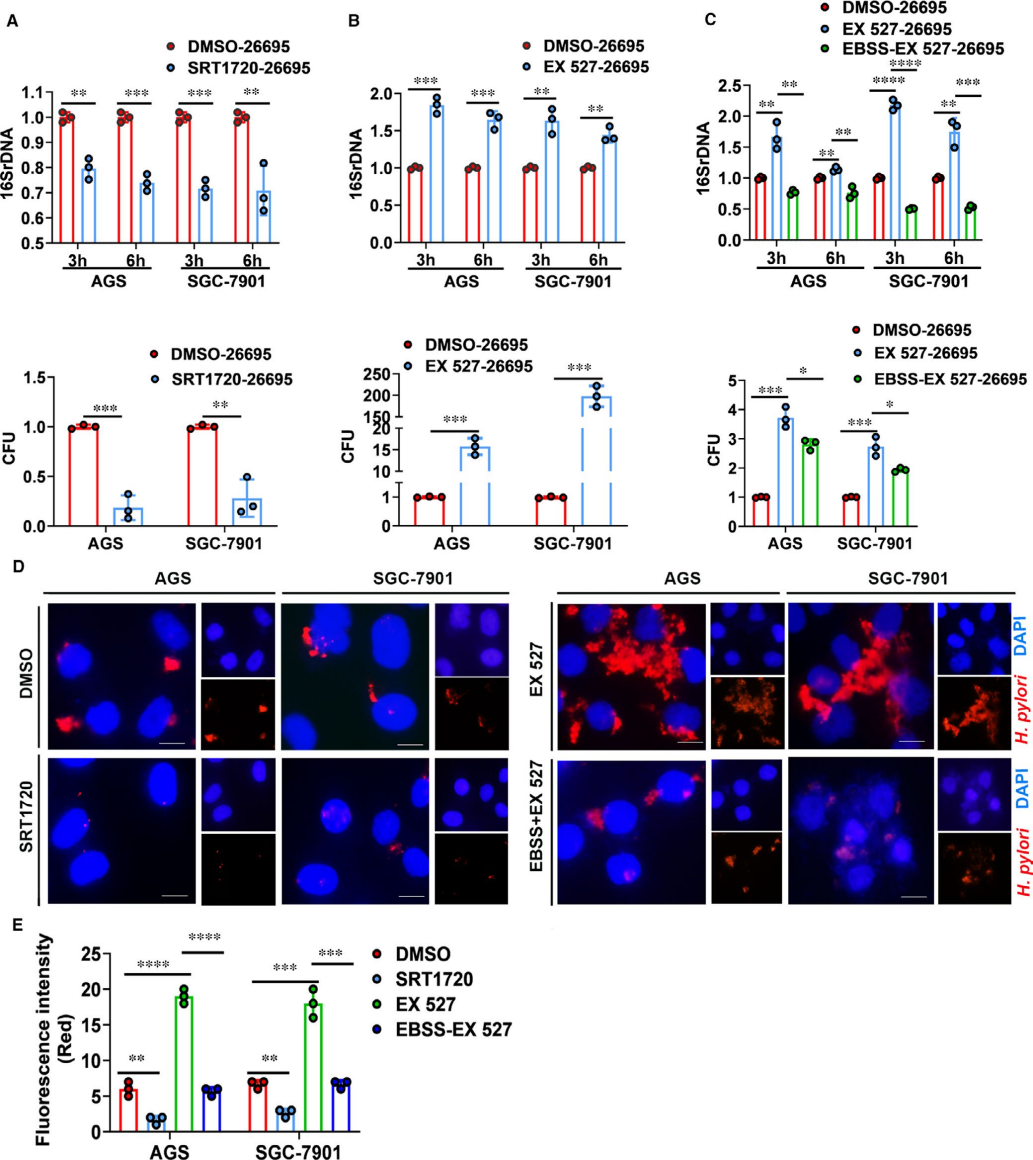
SIRT1 inhibits intracellular colonization of *H. pylori* by activating autophagic flux. A‐C, Detecting 16S rDNA and colony formation assays for bacterial quantitation. After pretreatment with SRT1720 (SIRT1 activator, 5 µM, 1 h) (A) or EX 527 (SIRT1 inhibitor, 5 µM, 1 h) (B), cells were infected with *Hp26695* and then used for further analysis. For rescuing experiments, EBSS (12 h) was used to induce autophagic flux in cells (C). Data from 3 independent experiments are presented as mean ± SD. D and E, Immunofluorescence staining of *H. pylori*. Cells were treated with SRT1720, or EX 527 or EBSS together with EX 527 as described in (A‐C). Data from 3 independent experiments are presented as mean ± SD. * represents *P* < 0.05, **represents *P* < 0.01, *** represents *P* < 0.001 and **** represents *P* < 0.0001

Then we investigated the effects of SIRT1 on intracellular survival and colonization of *H. pylori*. We pretreated *H. pylori*‐infected cells with SIRT1 activator (SRT1720) and then determined intracellular survival and colonization of *H. pylori* by bacterial DNA measurement, colony formation assays and immunofluorescence staining. As shown in Figure 6A, upon SIRT1 activation, gastric cells infected with *Hp26695* showed significantly decreased 16S rDNA levels and less colonies on agar plates indicating reduced *H. pylori* colonization. Additionally, immunofluorescence staining of *H. pylori* demonstrated less *H. pylori* colonization pretreated with SIRT1 activator (Figure 6D,E). To further confirm the effect of SIRT1 on *H. pylori* colonization, we pretreated *H. pylori*‐infected cell with SIRT1 inhibitor (EX 527). In contrast, SIRT1 inhibitor led to increased colonization of *H. pylori* in gastric cells (Figure 6B,D,E). Combined with our results that *H. pylori* infection inhibited expression levels of SIRT1, we demonstrated here that *H. pylori* infection inhibited SIRT1 expression and thus promoted its colonization in gastric cells. Next, we assessed whether the increase in *H. pylori* colonization can be reversed by autophagic flux. Serum starvation was used to induce autophagic flux in SIRT1 inhibitor‐treated gastric cells. Results from bacterial quantitation and immunofluorescence staining experiments showed that activation of autophagic flux significantly reduced the intracellular load of *H. pylori* in SIRT1‐inhibited cells (Figure 6C‐E). In brief, inhibition of SIRT1 increased intracellular survival and colonization of *H. pylori* which can be reversed by activation of autophagic flux.

### 3.7 Validation of relationship between SIRT1 expression levels and *H. pylori* infection in clinical specimens

**FIGURE 7:**
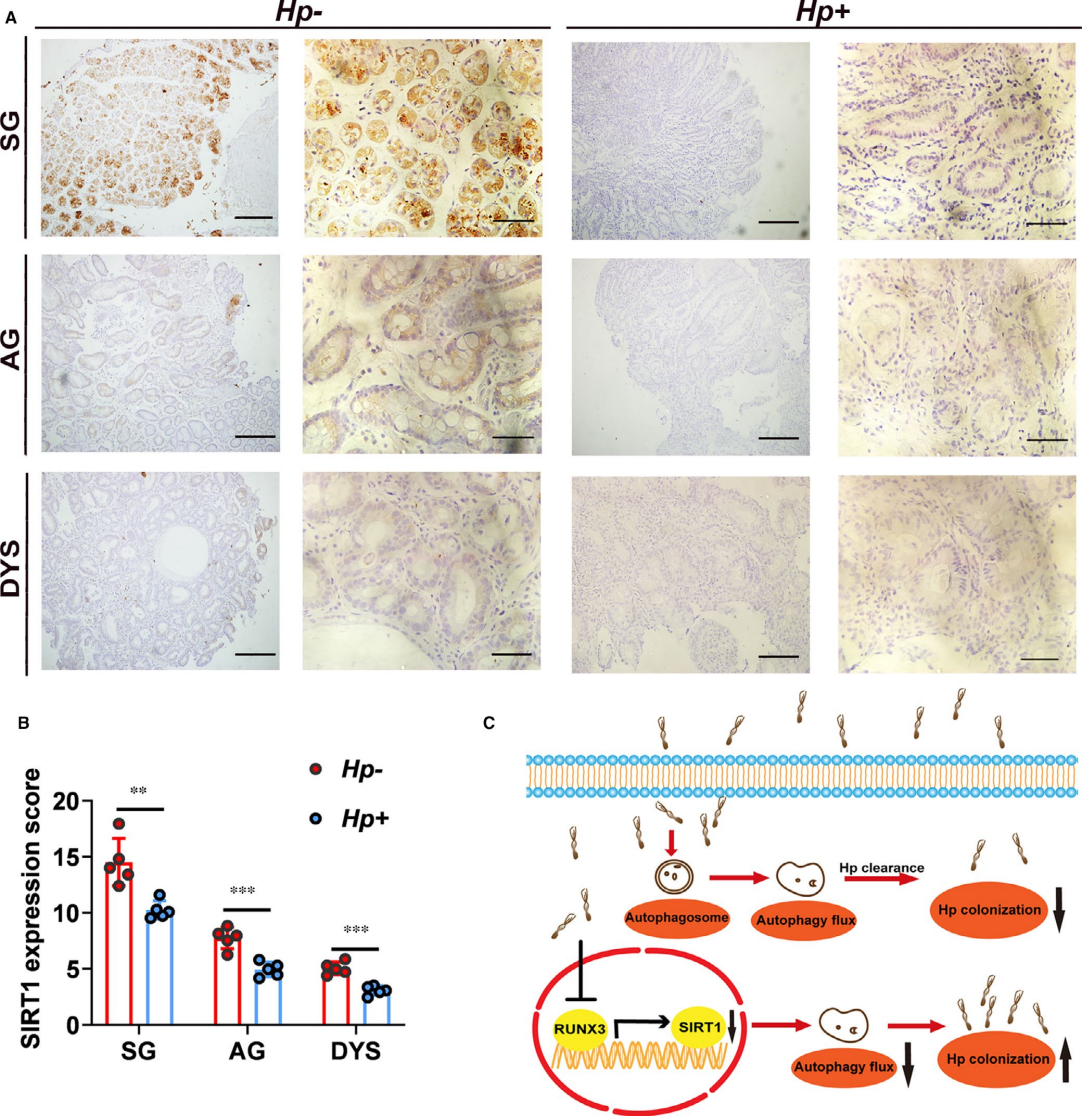
Validation of relationship between SIRT1 expression levels and *H. pylori* infection in clinical specimen. A, Representative images of IHC staining of SIRT1 in human superficial gastritis (SG), atrophic gastritis (AG) and dysplasia (DYS) samples. Scale bars: 200 µm for the left panel and 50 µm for the right panel. B, Statistics of IHC score of SIRT1 in clinical specimen. Comparison of SIRT1 expression score according to status of *H. pylori* infection. Data are presented as mean ± SD, n = 5. ** represents *P* < 0.01 and *** represents *P* < 0.001. C, Schematic model of study. Autophagy is a conserved protective process which can degrade intracellular pathogens such as *H. pylori*. Nevertheless, infection of *H. pylori* down‐regulated SIRT1 expression, inhibited autophagic flux in gastric cells and thus promoted its intracellular survival and colonization. Moreover, *H. pylori* infection inhibited expression of SIRT1 in a RUNX3‐dependent manner

To determine the relationship between SIRT1 expression levels and *H. pylori* infection, we performed IHC staining using clinical specimens. We collected gastric tissues diagnosed as superficial gastritis, atrophic gastritis and dysplasia. For each pathogenic stage, both *H. pylori*‐positive and *H. pylori*‐negative specimens were included. For each histologic stage (superficial gastritis, atrophic gastritis or dysplasia), expression levels of SIRT1 in *H. pylori*‐positive patients were significantly lower than those in patients *H. pylori*‐negative (Figure 7A,B). These results confirmed the relationship between SIRT1 expression and *H. pylori* infection in clinical specimen.

## 4 DISCUSSION

*H. pylori* is the strong risk factor for a series of gastric disorders, including gastritis and gastric cancer. Failure of our immune system to eliminate this bacterium leads to persistent colonization of *H. pylori* in gastric tissues and chronic infection. On the other hand, antibiotic treatment often cannot achieve a satisfactory result which makes it hard for *H. pylori* eradication.^25^, ^26^ Besides standardizing treatment regimen, exploring the mechanism underlying *H. pylori* colonization is critical to eradicate bacterial infection. In this study, we show that autophagic flux plays an important role in eliminating intracellular survival and colonization of *H. pylori*. Autophagy has emerged as an essential protective strategy employed by the host to eliminate intracellular bacteria since 2004.^27^, ^28^ On the other hand, intracellular bacteria develop diverse strategies to reprogram or stall autophagy process and thus escape from autophagic degradation.^29^, ^30^, ^31^, ^32^, ^33^ *H. pylori,* which was previously thought only survive on the surface of gastric mucosa, have recently been found to invade epithelial cells and colonize intracellularly. Although the intracellular part represents a small percentage of overall bacteria, this proportion is strongly resistant to antibiotic treatment and play a critical role in infection recrudescence post‐therapy.^3^, ^8^, ^9^ Here, we show that in gastric cells, *H. pylori* infection induced formation of autophagosomes but failed to induce autophagic flux. And the inhibited autophagic flux helps *H. pylori* to colonize inside the cells successfully.

Until now, the mechanisms underlying that intracellular bacteria initiate autophagy have not been completely elucidated. Nevertheless, compelling evidence suggests that intracellular pathogens may trigger nutritional deprivation and energy stress both of which were generally regarded as initial factors of autophagy.^29^, ^33^, ^34^, ^35^ SIRT1, a NAD^+^‐dependent deacetylase, acts as an energy and nutrition sensor. It has been shown that SIRT1 plays an essential role in calorie restriction‐induced longevity and starvation‐activated autophagy.^36^, ^37^, ^38^ By treating gastric cells with SIRT1 activator or inhibitor, we showed that SIRT1 can induce not only autophagosomes formation but also autophagic flux occurrence. The following rescue experiment indicated that activation of SIRT1 restored the autophagic flux inhibited by *H. pylori* infection. Through deacetylation of autophagy‐related proteins and promotion of phagosome‐lysosome fusion, activation of SIRT1 induces effective antimicrobial responses against mycobacterial infection and significantly reduces intracellular growth of both drug‐susceptible and drug‐resistant strains.^21^, ^23^ Consistent with the data of *M. tuberculosis* infection, by bacterial quantification and immunofluorescence staining, we demonstrated the inhibitory effects of SIRT1 on intracellular colonization of *H. pylori*. Furthermore, stimulation of autophagic flux by serum starvation significantly reduced *H. pylori* colonization in SIRT1‐inhibited gastric cells. Therefore, our findings indicate that the inhibitory effects of SIRT1 on *H. pylori* colonization are autophagy‐dependent. And these results collectively raise the possibility that activation of SIRT1 may serve as a potential therapeutic target in eradicating *H. pylori* infection.

*H. pylori* infection may induce complicated changes in gastric epithelial cells. Previous studies have found that *H. pylori* infection could increase SIRT2 expression levels whereas decrease SIRT3 expression levels.^39^, ^40^ Here using *H. pylori*‐infected cell models, we showed that *H. pylori* infection significantly inhibited expression levels of SIRT1 at both mRNA and protein levels. Next, we identified tumour‐suppressive transcriptional factor RUNX3 could regulate expression levels of SIRT1 in gastric cells. It has been revealed that *H. pylori* infection can inhibit expression of RUNX3 at mRNA levels through CpG island methylation or Src/MEK/ERK and p38 MAPK pathways.^41^, ^42^ Work from Tsang et al. even showed that *H. pylori* CagA targets RUNX3 for proteasome‐mediated degradation.^43^ The cytoplasmic mislocation of RUNX3 induced by *H. pylori* infection can also suppress transcriptional activities of RUNX3.^44^ Although we did not go further into the mechanism underlying *H. pylori* infection regulating RUNX3 expression, we identified down‐regulation of RUNX3 at both mRNA and protein levels after *H. pylori* infection. Overexpressing RUNX3 in *H. pylori*‐infected cells restored expression levels of SIRT1 suggested that inhibition of SIRT1 expression by *H. pylori* infection is RUNX3‐dependent.

In summary, we demonstrated that *H. pylori* infection induces autophagosomes formation but suppresses autophagic flux. *H. pylori* infection inhibited SIRT1 expression at both mRNA and protein levels. And transcriptional factor RUNX3 was identified to account for down‐regulation of SIRT1 by *H. pylori* infection. Inhibition of SIRT1 leads to decrease in autophagic flux induced by *H. pylori* infection and thus increased intracellular colonization of *H. pylori*. We further confirmed inhibition of SIRT1 expression by *H. pylori* infection in clinical samples. Our results provide evidence that SIRT1 can serve as a therapeutic target to eradicate *H. pylori* infection (Figure 7C).

## CONFLICT OF INTEREST

The authors declare no conflict of interest.

## AUTHORS CONTRIBUTIONS

Xin Wang, Bo Wang and Qing Yang: Study conception and design, Xin Wang, Bo Wang, Wei Gao, Yifei An and Guoying Dong: Performing the experiments, Xin Wang, Jihui Jia and Qing Yang: Data analysis and interpretation, Jihui Jia and Qing Yang: Writing the manuscript. All the authors had final approval of the submitted version.

## Supporting information

Fig S1

Fig S2

Fig S3

Table S1

Supplementary Material

## Data Availability

The data that support the findings of this study are available from the corresponding author upon reasonable request.
