# Supplementary figures and images for: Helicobacter pylori inhibits autophagic flux and promotes its intracellular survival and colonization by down‐regulating SIRT1
Source: J Cell Mol Med. 2021 Feb 28;25(7):3348–60. doi: 10.1111/jcmm.16411 (PMC8034483; doi:10.1111/jcmm.16411)

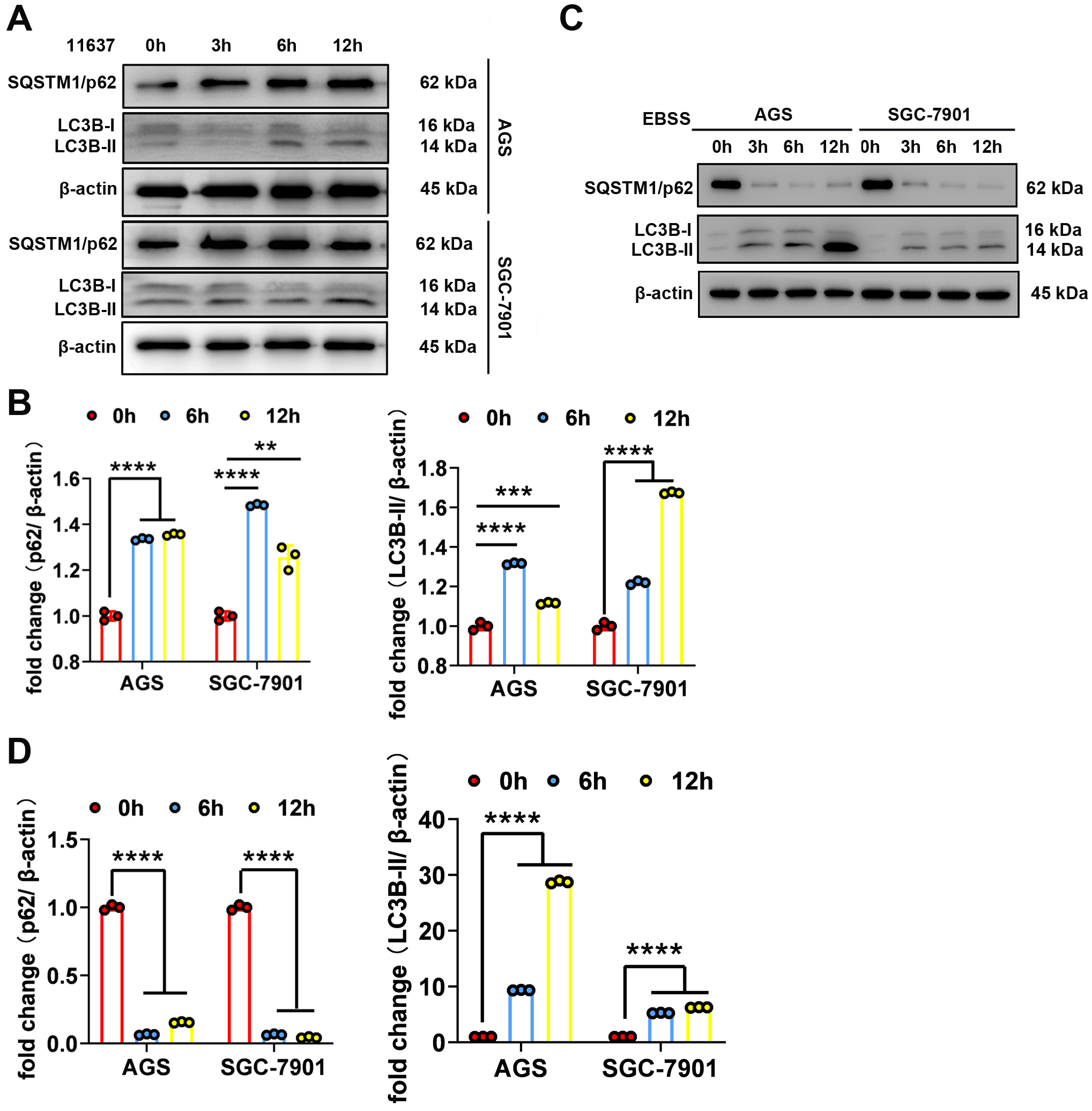

Supplement: Supplementary file 1 — Fig S1 [file JCMM-25-3348-s005.tif]

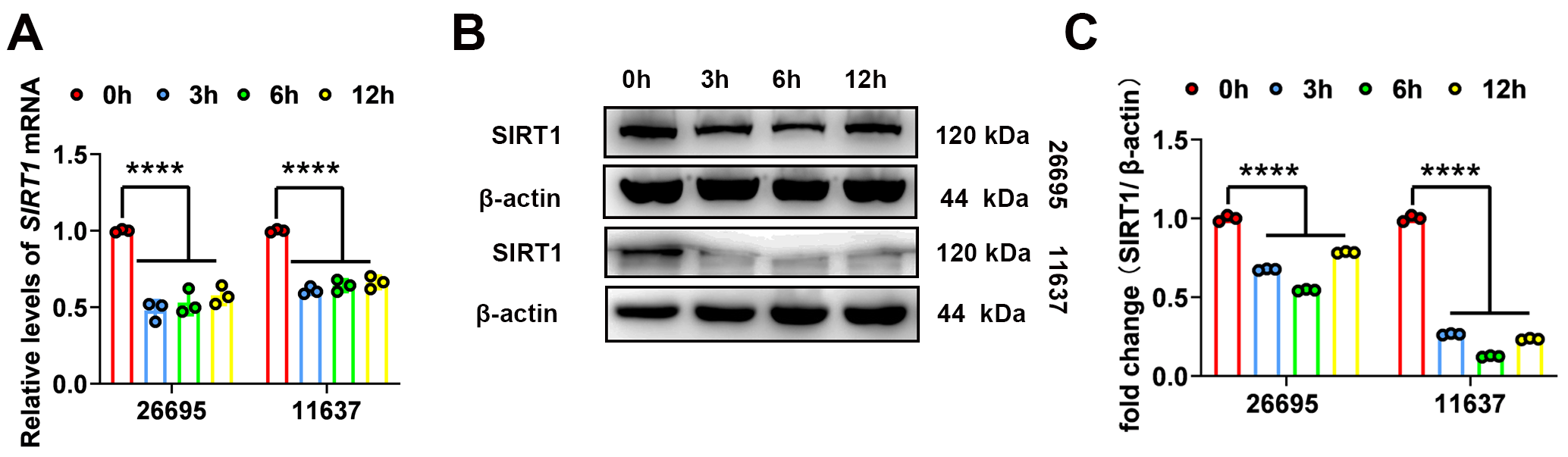

Supplement: Supplementary file 2 — Fig S2 [file JCMM-25-3348-s002.tif]

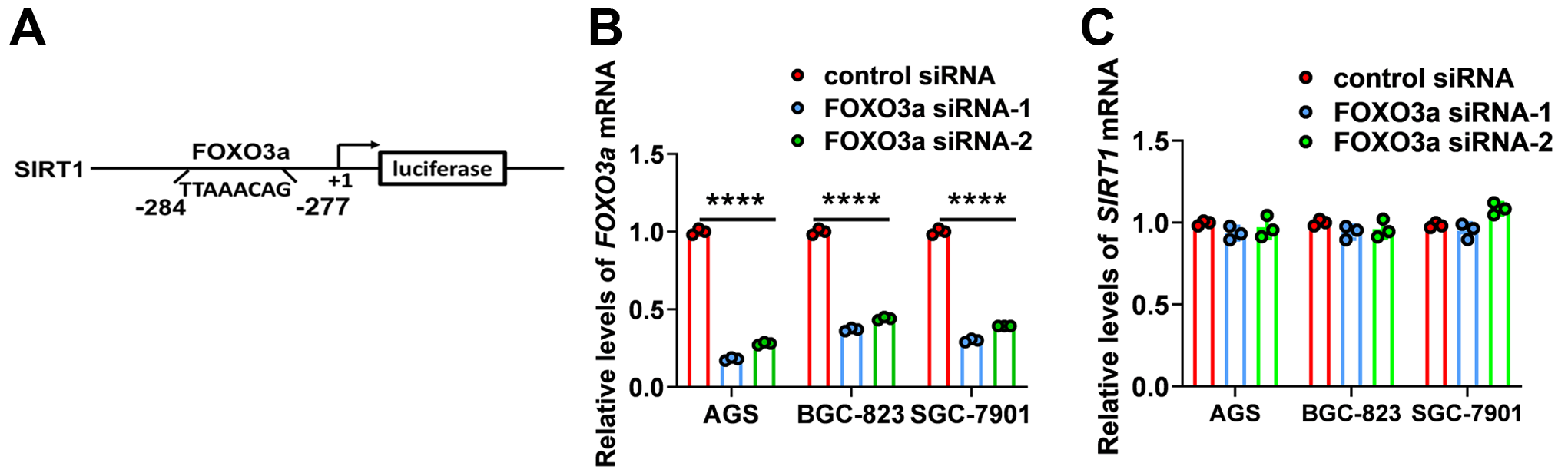

Supplement: Supplementary file 3 — Fig S3 [file JCMM-25-3348-s001.tif]
