# Supplementary material for: Helicobacter pylori inhibits autophagic flux and promotes its intracellular survival and colonization by down‐regulating SIRT1
Source: J Cell Mol Med. 2021 Feb 28;25(7):3348–60. doi: 10.1111/jcmm.16411 (PMC8034483; doi:10.1111/jcmm.16411)
Supplement: Supplementary file 4 — Table S1 [file JCMM-25-3348-s004.doc]

**Table S1.** Sequences of siRNAs and primers used in this study.

|  | **Gene** | **Sequence (5’ → 3’)** |
| --- | --- | --- |
| **siRNA** | Negative control  *RUNX3-1* | UUCUCCGAACGUGUCACGUTT  AGUUCUCGUCAUUGCCUGCCAUCAC |
|  | *RUNX3-2*  *FOXO3a-1*  *FOXO3a-2* | UGAAGUGGCUUGUGGUGCUGAGUGA  CCUCAUCUCCACACAGAAUTT  GCUCACUUCGGACUCACUUTT |
| **Primers** | *Sirt1*(*mouse*) | ATGACGCTGTGGCAGATTGTT  CCGCAAGGCGAGCATAGAT |
|  | *SIRT1*(*human*) | TGGCAAAGGAGCAGATTAGTAGG  CTGCCACAAGAACTAGAGGATAAGA |
|  | *RUNX3* | TACGGTGGTGACTGTGATGG  TGGCTTGTGGTGCTGAGTG |
|  | *16S rDNA*  *β-actin*(*mouse*)  *β-actin*(*human*)  *GAPDH(human)*  *Cre*    *flox*  *SIRT1* promoter  (*RUNX3* binding site) | GCTACAATGGGGTGCACAAA  TGAGTACAAGACCCGGGAAC  GGCTGTATTCCCCTCCATCG  CCAGTTGGTAACAATGCCATGT  TTGCCGACAGGATGCAGAA  GCCGATCCACACGGAGTACT  CTGCAGGTTCTCCACACCTATG  GAATTTGCCGTGAGTGGAGTC  GCCTGCATTACCGGTCGATGC  CAGGGTGTTATAAGCAATCCC  GGTTGACTTAGGTCTTGTCTG  CGTCCCTTGTAATGTTTCCC  ACTTATAGGTGAGCCATGGTGGTTT  CTGTAATCCCAGCTACTCAGAAGGC |
