# Supplementary material for: Helicobacter pylori inhibits autophagic flux and promotes its intracellular survival and colonization by down‐regulating SIRT1
Source: J Cell Mol Med. 2021 Feb 28;25(7):3348–60. doi: 10.1111/jcmm.16411 (PMC8034483; doi:10.1111/jcmm.16411)
Supplement: Supplementary file 5 — Supplementary Material [file JCMM-25-3348-s003.docx]

**Figure S1.** *H. pylori* (*Hp11637*) infection inhibits while EBSS promotes autophagic flux in gastric cells. Western blot was performed to detect the protein levels of LC3BI/II and SQSTM1/p62 in cells infected with *Hp11637* at an MOI of 100 (A, B) or cells treated with EBSS (serum starvation) (C, D) for indicated time. Data from 3 independent experiments are presented as mean ± SD. ** represents *p* < 0.01, *** represents *p* < 0.001 and **** represents *p* < 0.0001.

**Figure S2.** Examination of SIRT1 expression in *H. pylori*-infected GES-1 cells. (A) The qRT-PCR analysis of SIRT1 mRNA levels in GES-1 cells infected with *Hp26695* or *Hp11637*. (B, C) Western blot analysis of SIRT1 protein levels in GES-1 cells infected with *Hp26695* or *Hp11637*. Data from 3 independent experiments are presented as mean ± SD. **** represents *p* < 0.0001.

**Figure S3.** FOXO3 does not regulate expression of SIRT1 in gastric cells. (A) The scheme of the putative FOXO3-binding site in the SIRT1 promoter region. (B, C) The qRT-PCR analysis of FOXO3a (B) and SIRT1 (C) mRNA levels in cells transfected with siRNAs targeting FOXO3a. Data from 3 independent experiments are presented as mean ± SD. **** represents *p* < 0.0001.
